# Digital Biomarkers in Multiple Sclerosis

**DOI:** 10.3390/brainsci11111519

**Published:** 2021-11-16

**Authors:** Anja Dillenseger, Marie Luise Weidemann, Katrin Trentzsch, Hernan Inojosa, Rocco Haase, Dirk Schriefer, Isabel Voigt, Maria Scholz, Katja Akgün, Tjalf Ziemssen

**Affiliations:** Multiple Sclerosis Center Dresden, Center of Clinical Neuroscience, Department of Neurology, University Clinic Carl-Gustav Carus, Dresden University of Technology, Fetscherstrasse 74, 01307 Dresden, Germany; Anja.Dillenseger@uniklinikum-dresden.de (A.D.); MarieLuise.Weidemann@uniklinikum-dresden.de (M.L.W.); katrin.trentzsch@uniklinikum-dresden.de (K.T.); hernan.inojosa@uniklinikum-dresden.de (H.I.); Rocco.Haase@uniklinikum-dresden.de (R.H.); dirk.schriefer@uniklinikum-dresden.de (D.S.); Isabel.Voigt@uniklinikum-dresden.de (I.V.); maria.scholz@uniklinikum-dresden.de (M.S.); Katja.Akguen@uniklinikum-dresden.de (K.A.)

**Keywords:** multiple sclerosis, digital biomarkers, digital health technology, eHealth, precision medicine, personalized therapy, big data, digital twin

## Abstract

For incurable diseases, such as multiple sclerosis (MS), the prevention of progression and the preservation of quality of life play a crucial role over the entire therapy period. In MS, patients tend to become ill at a younger age and are so variable in terms of their disease course that there is no standard therapy. Therefore, it is necessary to enable a therapy that is as personalized as possible and to respond promptly to any changes, whether with noticeable symptoms or symptomless. Here, measurable parameters of biological processes can be used, which provide good information with regard to prognostic and diagnostic aspects, disease activity and response to therapy, so-called biomarkers Increasing digitalization and the availability of easy-to-use devices and technology also enable healthcare professionals to use a new class of digital biomarkers—digital health technologies—to explain, influence and/or predict health-related outcomes. The technology and devices from which these digital biomarkers stem are quite broad, and range from wearables that collect patients’ activity during digitalized functional tests (e.g., the Multiple Sclerosis Performance Test, dual-tasking performance and speech) to digitalized diagnostic procedures (e.g., optical coherence tomography) and software-supported magnetic resonance imaging evaluation. These technologies offer a timesaving way to collect valuable data on a regular basis over a long period of time, not only once or twice a year during patients’ routine visit at the clinic. Therefore, they lead to real-life data acquisition, closer patient monitoring and thus a patient dataset useful for precision medicine. Despite the great benefit of such increasing digitalization, for now, the path to implementing digital biomarkers is widely unknown or inconsistent. Challenges around validation, infrastructure, evidence generation, consistent data collection and analysis still persist. In this narrative review, we explore existing and future opportunities to capture clinical digital biomarkers in the care of people with MS, which may lead to a digital twin of the patient. To do this, we searched published papers for existing opportunities to capture clinical digital biomarkers for different functional systems in the context of MS, and also gathered perspectives on digital biomarkers under development or already existing as a research approach.

## 1. Introduction

Multiple sclerosis (MS) is a complex and chronic neurological disease of the central nervous system (CNS) that is characterized by a pathophysiological combination of neuroinflammation and neurodegeneration. As the inflammatory and neurodegenerative process can involve a variety of different neuroanatomical locations in the CNS, many functional neurological systems can be affected, ranging from visual, motor, cerebellar and sensory problems to complex cognitive symptoms. Since MS already occurs early in adulthood, accompanied by only a mildly reduced life expectancy, the highly heterogeneous disease, lasting over several decades, offers numerous inter-individually and intra-individually differences as well as different disease phenotypes evident in different disease stages [1]. Each of these individual differences and disease phenotypes must be addressed when it comes to treating MS as well as MS-related symptoms (e.g., spasticity, pain and gait problems). Additionally, as MS and its symptoms can change over time, it is crucial to detect these changes early in their development by using regular neurologic evaluation, questionnaires, functional tests, magnetic resonance imaging (MRI), laboratory checks and other assessments. Therefore, during this lifelong, chronic disease, a large amount of medical data accumulates, with important information pertaining to medical conditions and symptoms as well as diagnostic and therapeutic measures. In particular, the assessment of responders and non-responders to immunomodulatory therapies requires the long-term monitoring of different MS-related parameters, such as, for example, imaging, clinical assessments and biomarkers. If one adds the characterization of all different MS symptoms (e.g., depression and fatigue), the necessity for the complex and comprehensive collection of additional data becomes clear [1,2]. Since the collection of these data requires a lot of time, personnel and funds, using digital technology devices can facilitate this process and lead to the collection of so-called digital biomarkers. In this narrative review, we provide an overview of emerging digital biomarkers in the field of MS, their integration into regular monitoring and interesting approaches already in the testing phase, highlighting the need and benefits for the care of people with MS (pwMS). Searching for relevant literature in PubMed, specifying “digital biomarkers AND multiple sclerosis”, showed to be inefficient. Therefore, we decided to search for different functional systems with better results, although results in connection with multiple sclerosis are limited. Much more research has been done with digital biomarkers in other diseases, e.g., Alzheimer’s disease or depression [3,4,5]. Some of these digital biomarkers are now being investigated for their potential use in MS.

## 2. Digital Biomarkers

### 2.1. Definition of Digital Biomarkers

According to the National Institutes of Health (NIH, USA), biomarkers are objectively measured indicators of physiologic processes, pathologic processes or pharmacologic responses to a therapeutic intervention [6,7]. In MS, they can be subdivided into diagnostic (help to differentiate between different diseases, e.g., anti-aquaporin-4 antibodies, oligoclonal bands, etc.), prognostic (enable physicians to estimate how a disease might develop once it has been diagnosed, e.g., neurofilaments, oligoclonal bands, etc.), predictive (predict the treatment response and thus help to decide which patient is most likely to benefit from a certain treatment), disease activity (measure the inflammatory/neurodegenerative components of the disease, e.g., MRI, clinical parameters, etc.) and treatment response (responders versus non-responders of a certain treatment) biomarkers [6]. Especially with the focus on personalized medicine in pwMS, treatment response biomarkers can enable neurologists to differentiate patients regarding efficacy (e.g., neurofilament light chains, neutralizing antibodies against interferon-ß or natalizumab) or potential side effects (e.g., anti-varicella zoster virus antibodies, anti-John Cunningham virus antibodies) of a certain treatment [6]. The collection of such data is crucial to adapt the treatment of each patient individually to his/her results. However, it is also time-consuming if these data have to be gathered by physicians or other healthcare staff. With the increasing digitalization of healthcare, medicine now gains access to a new type of biomarker. So-called digital biomarkers enable the translation of up-to-date new data sources into informative, actionable knowledge. They can be used by healthcare professionals (HCPs) by implementing digital devices in their assessment (e.g., MRI, optical coherence tomography (OCT) and tablet-based neurostatus); they also enable data collection directly from the patient. They can collect such data directly as part of disease management on a regular basis, and thus ensure good monitoring and a prompt reaction to the progression of MS and the worsening of symptoms. Digital biomarkers mean objective, quantifiable physiological and behavioral data that are measured and collected by digital devices. The data collected by, e.g., portables, wearables, implantables or digestibles are typically used to generate, influence and/or predict health-related outcomes, and thus represent deep digital phenotyping, collecting clinically meaningful and objective digital data [8]. As digital technologies are usually less expensive than the process of collecting these data face to face, and as some of these data can be collected even without patients being actively involved (passive monitoring, e.g., by the use of wearables) data can also be collected more frequently and longitudinally. Health-related outcomes can vary, from explaining health and disease states, predicting drug responses or influencing health behaviors. In addition to this rather strict definition of digital biomarkers, digitalization in medicine also includes patient-reported measures (e.g., survey data), genetic information and other data that now can be collected by digital infrastructure. These data can complement the mentioned digital biomarkers, creating a digital multidimensional dataset.

Due to the technological transformation of healthcare, new technologies are leveraged to generate, track and collect new data. With the wealth of novel data, the responsibility is on the system to turn them into promising information that helps clinicians, researchers, patients and entrepreneurs to better understand states of disease and health [9].

### 2.2. Challenges of Digital Biomarkers

The path to implementing digital biomarkers in the clinic is complex, because the benefits that can be achieved by the use of digital biomarkers come with significant challenges (Table 1).

Digital biomarkers will, at least, face the same regulatory requirements as traditional biomarkers, and need to be tested for feasibility and reliability. The knowledge on how to establish and validate digital biomarkers is still limited. It can be challenging to identify relevant data and analyze them, and especially difficult in terms of how to use accurate baselines to relate this data for evaluation [10]. On the other hand, collecting continuous real-time data out of the patient’s everyday life closes the data gap between visits, and thus can reveal changes in the disease course as soon as they occur. A continuous dataflow from patients to their treating physician could generate a big dataset that shows real-world evidence, therefore being more meaningful and enabling faster decision making. This is only possible with patients who are carefully educated about the need for such sensitive data and demonstrate appropriate adherence. To avoid patients getting obsessed with even minor, non-significant changes, as to decrease the potential of over-reactions and increased anxiety, networking between physician and patient is crucial to evaluate and discuss the significance of these biomarkers. Besides necessary reflections on data security and the possibility to store these data over a long period of time, a huge dataset arises through the use of digital devices, which requires complex analyses.

Digital biomarkers have great potential for medical domains that are not well-understood, especially if digital biomarkers lead the way to phenotypic signatures. Challenges around infrastructure, evidence generation, consistent data collection and workflow remain.

To be seen less as a challenge than as an aspect to be considered is the distribution and availability of digital devices for data collection. Not every patient can afford to buy wearables or a smartphone to collect their data during their everyday life. In addition, some patients will have difficulties with their usage, due to age-related reasons or impairments that prevent the handling of digital devices.

### 2.3. Classification of Digital Biomarkers

Digital biomarkers are basically collected by digital tools. A way to classify these measures focuses on what has been measured, and the added clinical value derived from that data. At this, measurements can be familiar, such as the measurement of blood pressure, or innovative, such as the continuous measurement of blood pressure. A known clinical value is one that is well-understood and has previously been validated., e.g., blood pressure can be used as an indicator of cardiovascular risk. Alternatively, the known measurement can additionally be used to detect a new finding, linking blood pressure to, e.g., major depression. These different digital biomarker categories will influence the level of evidence required for regulatory approval, validation and clinical implementation [7,9].

### 2.4. Clinical Digital Biomarkers in Multiple Sclerosis

**Figure 1:**
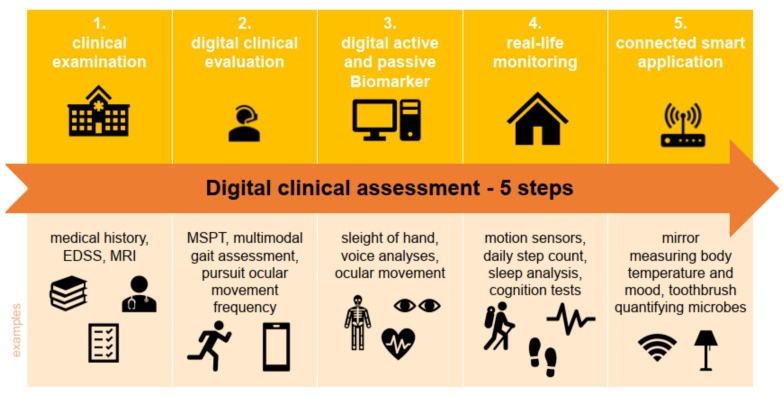
Developing a digital clinical assessment. (EDSS: Expanded Disability Status Scale; MRI: magnetic resonance imaging; and MSPT: Multiple Sclerosis Performance Test). © Multiple Scle-rosis Center Dresden.

Due to the increasing digitalization of health, a growing amount of patient data can be collected digitally in the care of pwMS (Figure 1). This not only refers to digital assessment results during clinical visits, but also daily patient-driven data collection, e.g., via the usage of smart devices, such as motion sensors, that arouse great interest in characterizing lifelong MS disease in a more granular way.

Figure 1 shows the five steps in digital clinical assessment from where we are now to where the future of digital clinical assessments could be. The typical clinical examination is still for the most part paper-based (except MRI, which is already digital), with, at best, subsequent digital storage of scanned documents in the hospital information system (step one). Digital clinical evaluations of, e.g., gait, patients’ perception regarding symptoms (patient-reported outcomes) or the digital version of the Multiple Sclerosis Functional Composite (MSPT; Section 3.4) are not available for every neurologic practice or hospital for use in clinical routine, but are available mostly as part of clinical trials (step two). Digital biomarkers cannot only be collected actively. Additionally, passive monitoring and data collection are possible using, e.g., voice analysis during calls with patients (step three). As step two relates to digital data collection at given points in time during patients’ visits, step three is already the transition towards data collection outside the clinical setting (e.g., passive collection of mobility via smartphones). Symptoms can vary over time, and disease progression may therefore be detected too late. For this reason, real-life monitoring is crucial (step four). Future devices could be smart applications, such as mirrors that automatically recognize body temperature and mood (step five).

Increasing evidence supports a forward-thinking chance of treatment decisions due to inter-individual highly variable clinical presentation, the extent of disease progression and a growing amount of defining biomarkers and surrogate endpoints, which personalize each disease presentation and favor our objective of a tailored treatment approach [1,11,12,13].

In the subsequent chapters, we will focus on digital biomarkers collected to investigate the involved functional systems or subdomains that are affected by different topographic lesions that occur during the course of MS. As MS is such a multidimensional disease, affecting different functional systems, collecting digital biomarkers capturing changes in those systems can offer insights into a comprehensively personalized disease.

## 3. Clinical Digital Biomarker by Functional Systems

### 3.1. Vision

Vision is one of the most affected functional systems in pwMS and often manifests itself in form of optic neuritis. Clinical signs can range from changes in color vision, reduced visual acuity or even complete loss of vision [14]. As atrophy of the retinal nerve fiber layer and ganglion cell layer was detected in 79% of pwMS and was 17 times higher in comparison to other neurological diseases, the measurement of the retinal nerve fiber layer can be used as a digital biomarker [15]. Using OCT, peripapillary retinal nerve fiber layer (pRNFL) thickness and macular volume can be measured to search for retinal atrophy [16]. Therefore, Martinez-Lapiscina et al. (2016) used models designed to determine the association of OCT-based metrics with the degree of disability, and included continuous variables such as pRNFL thickness as well as macular volume to quantify the effect (increase or decrease) on the risk of disability worsening associated with each unit of change (1 μm for pRNFL thickness and 1 mm^3^ for macular volume). Th results suggested that regular monitoring of the peripapillary retinal fiber layer could be a useful digital biomarker to monitor the worsening of disability in MS, especially as it correlated with clinical and paraclinical parameters of vision, disability and MRI [16,17].

Another digital biomarker that can be used to monitor vision impairment is contrast vision. Testing visual acuity at low contrast ratios is significant, because in pwMS the threshold at which a letter can still be distinguished from the background is significantly higher than in healthy persons [18]. Sloan low-contrast letter acuity (LCLA) has been shown to correlate with MRI parameters and with OCT-detected retinal nerve fiber layer thickness [19]. The benefit of such contrast vision screenings is that they can also be used on mobile devices such as tablets or mobile phones, and can be easily done at home by patients themselves on a regular basis. How such a test can be put into practice is described in Section 4.1 in more detail.

Furthermore, virtual reality (VR)-based visual field testing may offer options for in-clinic and self-testing at home in the future. To date, VR vision testing is not ready to be counted as a digital biomarker because a “standard of care” test is missing. However, after completing VR training, MS patients presented promising improvements in cognitive and motor function [20].

### 3.2. Brainstem

Regarding brainstem functions for neurologists treating MS, oculomotor function and dysarthria are particularly suitable to be used as digital biomarkers.

**Oculomotor function evaluation:** Among the clinical signs of brainstem involvement, oculomotor disturbances are a common symptom, and often present early in the course of MS (such as in relapsing–remitting MS (RRMS)) [21]. The most frequently observed eye movement disorders are saccadic dysmetria (91%), internuclear ophthalmoplegia (68%), vestibulo-ocular reflex abnormalities and gaze-evoked nystagmus (36%) [22,23].

The development of eye-tracking technologies became more popular because these technologies offer the chance to obtain in-depth information about how people explore the world, indirectly provide insights into higher-order cognitive processes, e.g., preference, and investigate attentional deficits. Additionally, these technologies enable oculomotor insights from a medical point of view (e.g., kinematic of eye movement, frequency and metrics of saccades in addition to response latency) [24,25].

One option to analyze eye motor function is to measure the saccadic initiation time (SI time), which describes the time until an appropriate saccade appears, beginning with a central visual cue [26,27]. Because of its close connection with ocular nerve impairment, saccadic tests are popular and most frequently used to assess oculomotor function in MS [28,29]. To measure SI time, participants fixate a central cross, and after replacing the cross through an arrow they make saccadic eye movements towards periphery stars in the corner of a screen [27]. Nygaard et al. (2015) found that the SI time of RRMS patients was significantly longer compared to age- and gender-matched controls. The presence or absence of white matter or brainstem lesions between patients had no influence on the SI time. However, eye motor disturbances might be an early indicator for a disseminated MS [27]. Another study by Finke et al. (2012) found a significantly larger decrease in saccade peak velocity and amplitude in pwMS suffering from fatigue in comparison to non-fatigued pwMS as well as to healthy controls when performing a saccade fatigue task that lasted 10 min [30].

Further, the pursuit ocular movement (POM) frequency has been analyzed in patients with RRMS and secondary progressive MS (SPMS) by using a vision-based non-intrusive eye tracker [23]. In the study of De Santi et al. (2011), the POM frequency was significantly lower in pwMS compared with age- and gender-matched healthy controls. Interestingly, no relation between POM and the Expanded Disability Status Scale (EDSS) and no difference between RRMS and SPMS patients could be found [23].

Numerous studies have indicated that besides measuring oculomotor characteristics, eye-tracking tools reflect multifaceted cognitive information, contributing to the prediction of cognitive impairment and having the potential to assess disease progression even in the absence of aware clinical symptoms [28,31]. This opens the possibility to use these tracking tools to further develop diagnostic tools, and to use the results as digital biomarkers to evaluate disease progression and prognosis more precisely. By now, eye-tracking tools have been used to detect pathologic visuo-spatial viewing behavior in MS [32]. New approaches present short assessments to capture abnormalities of the oculomotor system, such as SONDA (Standardized Oculomotor and Neurological Disorder Assessment) which takes less than five minutes for the whole assessment and is also used in Parkinson’s disease [33]. Quick and standardized assessments allow regular monitoring over time without overburdening patients, especially those suffering from fatigue.

**Speech analysis:** Speech and voice are frequently impaired in MS, with a prevalence of approximately 40–50% [34,35], within which dysarthria is the most frequent communication deficit [35]. Its presentation usually tends to be mild, so unintelligible speech is very rare [36,37]. The major dysarthric features are deficient loudness control, slowness, monopitch, increase in pauses, strained voice, imprecise consonants and decreased respirator capacity [13].

However, it is important to consider that impairment of speech may have negative effects on social participation and employment status, resulting in an overall reduced quality of life [13]. So far, the basic characteristics of pwMS with dysarthria related to prosody and articulation remain mostly unresearched [38]. Accordingly, regular screening for changes in speech may contribute to the gain of important new biomarkers of disease progression, wherefore further developments in technology make the quantitative acoustic assessment of speech possible [13,38]. Digital vocal biomarkers offer the possibility of a standardized measurement and monitoring of speech. As speech might also be influenced by fatigue, depression and impairment in verbal cognition [38,39,40], the evaluation of speech also enables us to screen for these aspects and expands the spectrum of measurable parameters.

Studies showed a statistically significant correlation between dysphonic symptoms and MS, and the odds for having MS were 2.2 times higher if dysphonic symptoms were present with high jitter and shimmer values as well as high soft phonation index (an indicator of vocal fold adduction; high values correlate with incomplete adduction of the vocal fold [41]) values [42,43]. The objective acoustic analysis of speech seems to be more sensitive for discrimination between affected patients and healthy controls (90% accuracy) than experienced raters (35% accuracy) are, and thus could be used as a biomarker for diagnosis and the monitoring of disease progression [13,35,44,45].

Speech analysis applications using artificial intelligence to evaluate acoustic speech and language measures via a tablet are thought to provide vocal biomarkers for diagnosis, risk prediction and regular monitoring not only in MS but also in Parkinson’s or Alzheimer’s disease, and are in general highly predictive for cerebellar dysfunctions [46,47]. The benefit of a standardized software-based evaluation of speech tasks is the avoidance of intra- and inter-individual deviations in perceptions [47], so that monitoring could also be possible outside of routine visits. Digital vocal biomarkers and their use in clinical practice are still facing challenges when it comes to different accents, ages, task complexities and individual cognitive abilities [47,48]. Signs of fatigue and depression are already detectable in healthy individuals or patients without neurological disease [39,49]. As fatigue, depression and cognitive impairment are common in MS, they could be detected by speech analyses [46]. Test batteries can be designed in such a way that they capture executive functions and processing speed (e.g., phonematic and semantic word fluency [50]), memory (e.g., the Wechsler Memory Scale and California Verbal Learning Test [51,52]), affect and fatigue (e.g., storytelling [53]), language (picture description [54]) and motoric function (Pa-ta-ka task [38,42]). To date, performing such speech and language tests might be limited to trials, but can be imagined to be used in the future during clinical visits of pwMS or even at home by the use of specific apps or recordings during telemedicine visits.

### 3.3. Upper Extremity Motor Function

At least 56% of pwMS have upper extremity impairment; 71% of those report limitations in hand and arm use that dramatically affect daily living activities [55,56,57]. Upper extremity impairment is mostly conditioned by weakness and/or impaired coordination/ataxia [58], and is likely to limit future ability to perform activities of daily living and further reduce quality of life.

Existing dysfunction increases with disease progression, especially in patients with progressive MS compared to patients with RRMS [56,59]. Due to their highly differentiated movement variability, a comprehensive assessment of the upper limb can be challenging, and thus assessments must address multiple subsystems, such as eye–hand coordination and intra-limb and inter-limb coordination, as increasing dysfunction is seen in patients after stroke or with other diseases affecting the coordination of the limbs [57,60,61,62].

One of the most popular functional outcome measures to examine upper extremity function is the Nine-Hole Peg Test (9HPT). The 9HPT has known deficiencies—it only assesses fine manual dexterity; other important upper extremity functions, such as proximal upper extremity movements, complex bimanual tasks or the manipulation of larger objects, are not captured [63]. Accordingly, there is an ongoing search for new, multidimensional, sensitive upper extremity performance tests that provide new biomarkers that may predict disease progression. Here, the widespread use and manual handling of smartphones make them a promising assessment device, especially with regard to their ever-increasing abilities. With smartphones containing sensors, such as a gyrometer, accelerometer, inclinometer, orientation and light sensors, the opportunities to develop new ways to measure neurological functions seem almost infinite [64].

Tanigawa et al. studied finger tapping via a smartphone-based app as an alternative outcome measure of upper extremity function in MS by an analogy to tapping as a useful outcome measure, e.g., in primary lateral sclerosis [65]. Finger taps correlated clearly with 9HPT results. Furthermore, a correlation between tap results and other raised measures of physical disability could be shown [64].

Several smartphone-based apps capturing different functional systems via digital tests and questionnaires have emerged, including the Floodlight app and Konectom (see Section 4.1). These apps contain tests for the upper extremities and use assessments to capture more than just fine motor skills. The pinching of balloons or tomatoes that emerge on different positions on the screen or the tracing of a figure with the index finger of both hands as quickly and accurately as possible measures, e.g., eye–hand coordination, fine motor function and the pressure of the fingers on the screen as well. Creagh et al. analyzed pwMS and healthy controls tracing a predefined shape on a smartphone, demonstrating an authentic prediction of 9HPT results [66].

The use of such apps on patients’ smartphones enables a regular and continuous progression monitoring of upper limb function even outside of the clinic by patients’ themselves, without supervision [64]. However, in order to fulfill the function of a monitoring tool and to influence therapy decisions, it must also be ensured that the test results are transmitted to the treating physician.

Additionally, depth camera systems together with machine learning algorithms were examined to objectively quantify changes in movement-related symptoms to discriminate between healthy, not healthy and disease progression, which still needs to be researched further [67].

Another possibility with which to measure impairments in upper limb function are questionnaires (patient-reported outcome measure, PROM) that address different aspects of daily usage of hands and arms in different situations, such as tying shoes, buttoning up shirts or opening bottles. A regular questioning of the patient regarding his or her impairments provides important indications of upper limb dysfunction and potential further examination. To date, no standardized upper limb PROM has been established.

### 3.4. Lower Extremity Motor Function/Gait

Lower extremity impairment and the resulting gait deficits are the most frequent and visible consequences of MS, caused by a variety of pathophysiologic conditions such as pyramidal, cerebellar or sensory dysfunction [68]. Approximately 85% of pwMS report impaired walking, with an often profound impact on daily life [69,70]. Compared to healthy controls, abnormal gait characteristics of pwMS are characterized by decreased walking speed, shorter step and stride length, prolonged double limb support time and increased step variability [70,71]—even without clinical evidence of gait disturbance early in the course of the disease [72]. Several factors are thought to contribute to gait impairment in pwMS, of which sensory changes and the resulting imbalance, weakness of the lower limbs or spasticity and cerebellar ataxia might have the biggest impact [73]. Depending on one’s assessment goals, different tools can be used for the evaluation of gait impairment in pwMS, ranging from standardized clinical measures, timed measures, patient-reported outcomes, observational gait analysis, instrumented walkways or three-dimensional gait analysis, which all require different expertise of the examiner, time and equipment [73]. Each of them show advantages and disadvantages (Table 2).

In the following, these assessments are presented for the different settings of research, in-clinic monitoring assessment or functional tests to be performed at home, whereby the application of these tools is not always limited to one area. Wearables and smartphone apps, e.g., enable their use in several areas. As in 50% of pwMS with lower gait dysfunction also show upper limb impairment [76] this needs to be examined and addressed as well when therapy is considered.

#### 3.4.1. Lower Extremity Function in MS Research

Research offers the possibility to use more advanced technologies for movement analysis than in common standardized clinical assessments providing a higher sensitivity for subtle impairments [75]. Therefore, not only a complex infrastructure is needed but also trained medical staff to accompany pwMS and to conduct the tests as well as to analyze the data. A selection of potential assessment technologies in MS and a selection of their associated outcomes is shown below (Table 3) [74].

Video-based assessment technology captures so-called kinematics (motion sequences and range of motion) regarding time, place, speed and acceleration [77], either marker-based or marker-free. Marker-free systems show to be more user-friendly; however, marker-based systems are on the one hand more time consuming and involve extensive technological and human resources, but on the other hand offer higher accuracy and reproducibility [74,75].

Sensor floor plates allow for the measuring of spatiotemporal parameters (instrumented walkways such as GAITRite^®^), as well as information about ground reaction force (force platforms or balance boards). As such systems are mainly focused on muscle force, joint load and moment during initial contact and toe-off to evaluate gait impairment [78], other aspects of mobility are missed, such as swaying, rotation and balancing of the body, data which are needed to obtain a more precise movement pattern of pwMS. Therefore, all of these devices can be expanded by wearables.

Wearable sensors can also be used at home by patients themselves to measure their gait restrictions. One wearable for research use is the Mobility Lab system (APMD, Portland, OR, USA) which consists of Opal sensors that are fixed on specific body parts (e.g., wrist, sternum, lower lumbar spine and feet) [79]. Three-dimensional linear acceleration, angular velocity and magnetic field (for directional orientation) are captured by the use of onboard accelerometers, gyroscopes and magnetometers, and Mobility Lab software analyzes these data for gait parameters such as stride length, velocity, cadence, stand and swing time, etc. [79]. In addition, so-called consumer-driven wearables (e.g., GPS watches) are of interest as they can provide data collected in research, a clinical setting or at home.

Video-based and sensor floor plates assessments are only possible to perform in an in-clinic setting, whereas wearable sensors can also be used by pwMS themselves in their daily living over a longer period of time, thus providing an additional quantitative large dataset which better represents the mobility of pwMS. Spain et al. could also show that body-worn motion sensors could discriminate pwMS from healthy controls with a higher sensitivity than tests conducted using stopwatches, as wearables can also detect sway and axial rotation while the latter only captures speed, which might not show any impairment yet [80].

#### 3.4.2. Lower Extremity Function in the Clinic

The most frequently used clinical assessment tool and outcome measure in MS, the Expanded Disability Status Scale (EDSS), considers general ambulation by rating gait impairment upon endpoints, e.g., requirements of rest, dependency on help or loss of walking ability/wheelchair [81]. Thereby, subtle functional impairment cannot be taken adequately into account, leading to an insensitive scoring concerning disease progression, especially in the early stages of the disease [68,82]. However, as subtle gait impairment and balance dysfunction are seen as precursors of mobility loss in MS [75], the need for suitable outcome measures, capable to detect even subtle gait impairments and to monitor disease progression during a clinical assessment and also out of the artificial clinical setting under real-life conditions of pwMS becomes clear [72]. Nevertheless, it is also necessary to monitor the worsening of gait and balance dysfunction throughout the whole disease course. Interventions (pharmacologic and/or non-pharmacologic) need to be started and/or optimized as soon as possible to prevent further or faster progression of disability. One test alone is not able to describe impairment in the many facets of walking of pwMS. A combination of standardized functional tests that capture walking speed, walking endurance and balance as well as the quality of walking, or standardized patient reported outcomes regarding mobility restrictions will lead to a broad and sensitive dataset to evaluate mobility, at best on a regular basis. Various digital tools can be used for this purpose. Many of the gait assessments available in the research are not possible, as not everyone can be transferred into a routine clinical setting due to a lack of infrastructure, time, space or well-trained staff [74]. For those who can afford to integrate a broad evaluation of mobility into their clinic, all of the above-mentioned assessments can be performed. It is recommended to implement a protocol to follow and, thus, enable a standardized measurement for pwMS. At the Multiple Sclerosis Center Dresden, we developed the Dresden Protocol for Multidimensional Walking Assessment (DMWA) [74] to capture mobility in all of our pwMS at least once a year. Thus, with this long-term monitoring, early walking impairments as well as development over time or even the response to certain gait-influencing drugs can also be recorded, and the current standard of clinical practice be improved [74]. Gait analysis can also be supplemented in the clinical setting with other (digital) functional tests that include, e.g., cognition, speech, vision, or PROMs. Therefore, we added the Multiple Sclerosis Performance Test (MSPT) to the clinical routine of pwMS.

The MSPT is a tablet-based (iPad Air^®^ 2, Apple, Cupertino, CA, USA) digital assessment tool (app) designed to be used in a routine clinical setting without or with only minimal supervision [83]. Based on and extending the Multiple Sclerosis Functional Composite, the MSPT uses a digital adaption of the Symbol Digit Modalities Test (SDMT), the Sloan low-contrast visual acuity test, the 9HPT and the Timed-25 Foot Walk (T25FW) tests as well as a questionnaire regarding quality of life in neurologic diseases [58,84,85,86,87]. The MSPT includes all tasks to evaluate cognitive function (Processing Speed Test (PST), a digital adaption of the SDMT), contrast sensitivity, upper extremity function (9HPT) and walking speed/lower extremity function (T25FW) [83]. The aim is to assess the often-impacted neurologic functions of pwMS regularly and standardize them to create a longitudinal digital medical record, contributing to a better disease understanding and progression monitoring, which may contribute to more optimized patient care and management [83]. The MSPT is basically meant to be performed by pwMS without supervision, which allows data collection without consuming time and staff at the clinic. Data are available right after completion and can also be used for monitoring; therefore, a baseline MSPT should be performed at the time of diagnosis or treatment start/change to refer changes to. The benefit of using the MSPT is the availability of standardized functional testing and the possibility of having a great amount of pwMS performing the MSPT without the requirement of additional staff. Learning effects, such as for the paper-based SDMT [88], are excluded by randomly assigning numbers to symbols for every assessment [89]. Studies showed excellent test–retest reliability for the manual dexterity test (digital version of the 9HPT) and the walking speed test (T25FW), a significant (but only modest) correlation of the contrast seeing test with the standard Sloan low-contrast vision acuity [90] and an excellent test–retest reliability for the PST, with a high correlation with the SDMT and with cerebral T2 load (in contrast to the SDMT) [89].

The implementation of the tests in a digital format is user friendly as each test is explained by a video. The tablet is brought in an upright position. At first, information about current disease modifying therapy (DMT), relapses and Patient Determined Disease Steps is made before filling out the NeuroQoL [91,92,93]. The PST shows a random assignment of numbers to symbols and ten symbols at a time for which patients have to choose the correct number by tapping on one of the numbers shown at the bottom of the screen. For contrast vision, a certain distance and illumination are needed before the test can be started. At first, letters are shown at 100% and then at 2.5% contrast, whereas patients have to choose the letter they see out of a collection of letters at the bottom of the screen. In cases where a letter cannot be clearly identified patients can guess or tap “unclear”. To perform the 9HPT, the MSPT needs to be lying flat on the table and the stand with the pegboard is folded down. Nine pegs are put in the row at the bottom and after activating the countdown patients are asked to take one peg at a time from the row and insert it into one of the holes of the pegboard. After all pegs have been inserted, they need to be removed, again, one at a time, and put back into the row. Time automatically stops when the last peg is put in the row at the bottom of the pegboard. Before performing the T25FW patients need to specify if they will use any gait support or if they wear any lower leg orthosis. If patients are rather unsteady on their feet, a nurse supports the patient to avoid any falls. At the end, an overview of the results can be seen on a dashboard, and the longitudinal course can be seen by tapping each test.

Despite the fact that the MSPT was designed for pwMS to be performed without support or supervision, we recommend pwMS to be supported if needed, and the provision of feedback to their results increases adherence to and understanding of monitoring.

#### 3.4.3. Lower Extremity Function at Home

The collection of real-time data on a longitudinal basis becomes more and more important when monitoring chronic diseases in particular, such as MS, as they do not only state a condition at one point in time and thus allow for progress and follow-up control. In particular, accelerometers are used, and depending on their position on the body, allow for the partial documentation of the relevant mobility; however, not all physical activities are captured equally as well [94]. With the help of wearables, it is possible to focus on different aspects such as gait, upper or lower limb function, behavior or other body movement patterns; when used regularly, they provide information about mobility from outside a clinical setting and may correlate with disease-specific predictors, outcomes or interventions [95]. Various accelerometers can now be used, such as the already widely used fitness trackers (e.g., Fitbit, Garmin, Xiaomi, ActiGraph and others), which have been shown to be useful in an everyday setting and can even be used to collect data over several days [74,82,96]. By tracking the physical activity of pwMS continuously over one year, Block et al. (2019) could show an association between a reduction in average daily step count and the worsening of standard clinic-based and patient-reported metrics [97]. They also showed that patients with a lower baseline average daily step count were found to be at a higher risk of disability worsening one year later [97]. Other wearables, such as the skin-mounted inertial sensor BioStampRC (MC10, Inc., Cambridge, MA, USA), could support physicians in identifying gait pathology and in evaluating disability progression of gait in pwMS [98]. As wearables become more and more affordable and broadly accepted, they might work as an ambulatory, real-life and continuous gait monitoring system [98,99], allowing for increased sensitivity in regard to monitoring disease progression and the efficacy of immunomodulation [98].

Captured variables can include step count, active minutes, activity count, activity bouts and energy expenditure [97]. Compared to non-wearable laboratory/research systems, wearable sensors capture a smaller number of gait variables [75].

Smartphone-based apps that use functioning tests or record movement parameters are another way of tracking patients’ mobility and activity. They will be discussed in Section 4.1.

### 3.5. Coordination/Balance

Deficits in balance are, even in early disease stages, common [72,100,101,102]. Overall, 50–80% of pwMS state balance problems over the course of the disease [103,104]. Balance can be defined as a skill of the nervous system, using several systems such as passive biomechanical elements, all available sensory systems and muscles as well as a multitude of different parts of the brain, instead of simply reacting reflex-like to perturbations [105]. As the heterogeneous demyelinating lesions in MS could also affect somatosensory or vestibular paths, visual input was shown to be necessary to maintain postural control in pwMS [106]. Postural control is defined as the act of maintaining, achieving or restoring a state of balance during any postures or activities [107], which a person tries to achieve by reactive, predictive or a combination of both behaviors [108]. Postural control is closely associated with falls in pwMS [106], which emphasizes the need for longitudinal evaluation during the course of the disease. As for today, postural perturbations are subjectively rated by neurologists as part of the cerebellar functional score of the EDSS [109]. To avoid subjective judgment of postural control in MS and to allow for follow-up evaluation of changes, objective, digital and quantifiable measurements are needed. In a clinical or research assessment, proprioceptive deficits can be evaluated, e.g., by using the Romberg test. To objectify its results, it may be connected with the use of body sensors (e.g., Mobility Lab system) that allow software-based calculation of deviations from the norm [110]. Static posturography is another method of assessing balance in which patients are asked to stand on a force platform with their feet closed and their eyes closed or open for 20 to 60 s to measure spontaneous body sway, which can be extended by more difficult stand trials (e.g., tandem stand or standing on one leg) [111]. Balance parameters that can be captured include average sway and speed as well as delineated area [106]. Inojosa et al. (2020) showed in their study that static posturography could detect balance impairment even if patients had no disability according to their neurological examination [106]. Special apps for smartphones also provide tests with which to perform the Romberg test for balance evaluation and other gait assessments to be performed at home by pwMS themselves (see Section 4.1). Additionally, the use of portable balance boards (e.g., Nintendo Wii) are under investigation to be used as an inexpensive alternative to force platforms for balance assessment in pwMS [112]. An interesting aspect here is whether balance training could have a positive impact on postural control in pwMS. In their review on balance improvement, Gunn et al. reported a positive influence of exercise interventions on balance in pwMS [113]. Other studies focusing on general motor rehabilitation in pwMS pointed at the issue that motor learning consists of three stages (cognitive, associative and autonomous phase), where the first stage depends on the person’s cognitive abilities [114], and the fact that cognitive impairment is very common in pwMS thus connects cognitive and mobility dysfunction [115].

### 3.6. Cognition

Approximately 40–60% of pwMS report cognitive dysfunction, and it is not uncommon that the symptom onset is immediately after first disease manifestation or even before [116]. Impairment in cognition can occur at all stages of the disease and in all MS phenotypes [117]. Frequently impaired domains are working memory, verbal fluency, information processing speed, verbal and visual memory, executive functions [84,118] and, according to new findings, “the theory of mind domain” (the ability to conclude on the basis of nonverbal and verbal hints about other people’s emotions) [119]. As cognitive impairment is a strong predictor of health-related quality of life (QoL) [120] and QoL in turn has a huge impact on adherence [121], together with the negative impact of cognitive dysfunction on employment [122] and many other aspects of life [123], a thorough and regular evaluation is necessary [118]. So far, cognitive monitoring is often a not-well-established part of standard care in MS. This is partly due to time and staff that are needed to allow for a routinely, longitudinal follow-up of pwMS. Therefore, digitalization of cognitive assessments where patients are able to perform these by themselves without supervision can enable long-term cognitive monitoring. Provided in a smartphone-based format, this monitoring could be done also at home, e.g., with the Floodlight app (Roche, Basel, Switzerland) to perform the SDMT or the MS Sherpa app (Orimaki personalized healthcare, Nijmegen, The Netherlands) to evaluate the cognitive signal processing speed (see also Section 4.1).

Implementing digital cognitive assessments in the monitoring of MS is challenging, given the fact that many pwMS show not only cognitive deficits but also physical impairments that are required for this kind of testing and need to be addressed when transforming paper-based tests into a digital form, as they can change what exactly is measured [124]. For clinical use, a number of simplified tests of cognition have been developed in MS, including test batteries such as the Brief Repeatable International Cognitive Assessment for MS [125], the Brief Repeatable Battery of Neuropsychological Tests [126] and the Minimal Assessment of Cognitive Function in MS [127,128]. The transformation of such tests into a digital form (computerized neuropsychological assessment device (CNAD)) is considered very controversial by some experts, stating that this transformation results in a new and different test: it has a different patient interface and is also available to examiners with no expertise in neuropsychological assessments or knowledge of psychometric principles, and thus no accurate interpretation of test results is achieved as other factors influencing performance are not considered, no observational interpretation of the examinee is possible, etc. [129]. Other review papers could show, e.g., for the PST, the Computerized Speed Cognitive Test and Computerized SDMT (C-SDMT), compared with the SDMT, a high test–retest reliability and validity, and for other tests acceptable psychometrics [130]. Before applying CNADs to clinical routine or trials, adequate test–retest reliability and sensitivity should be demonstrated [130].

Amato et al. (2001) already showed that if a follow-up was long enough, cognitive dysfunction was likely to emerge in a great proportion of pwMS, re-emphasizing the need for regular, standardized monitoring [123]. It has been shown that assessing cognitive function early in the course of the disease did not only identify cognitive impairment in individuals but could also predict future impairments, limitations and MS disease progression [131]. Thus, recommendations can be made to start cognitive assessments right from the start and re-assess cognitive functions in pwMS, thus enabling early treatment interventions.

As interactions between motor and cognitive functions are known in MS, linking them together (termed dual-tasking) can be used to evaluate the interference of performing a cognitive task during gait assessment [73,74,132,133,134].

#### Dual-Tasking

**Figure 2:**
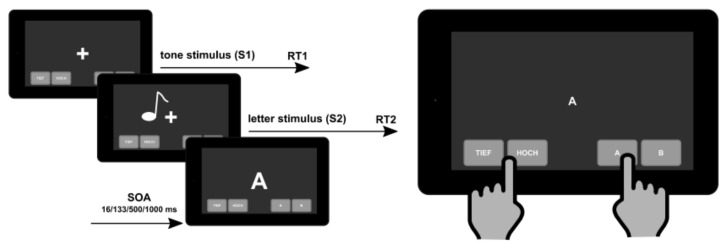
Illustration of the psychological refractory period paradigm as a dual-task assessment in MS. Stimulus 1 (tone) is always presented first, followed by stimulus 2 (letter) in a defined stimulus-onset asynchrony (SOA). PwMS are advised to respond first to stimulus 1 and as quickly as possible to stimulus 2 by pressing defined keys. RT: reaction time [147].

Coordinating two or more tasks simultaneously is an everyday requirement, and is increasingly recognized in the treatment and supervision of pwMS as having a major impact on employment status [135]. This makes it even more important to recognize early and subtle cognitive (executive) dysfunctions [136]. Up to now, dual-tasks are performed during walking or balancing and, e.g., showed a slowing of gait depending on MS disease severity [136,137,138,139,140,141,142,143,144]. Dual-task tests that are already able to detect subtle and early executive dysfunctions are still lacking in MS. A study investigated the use of a standard psychological refractory period (PRP) paradigm [145,146] in pwMS where two tasks (first stimulus—high or low tone; second stimulus—letter A or B) have to be performed which are presented in close succession and to which pwMS have to respond as quickly as possible (Figure 2) [136].

This dual-task test is still under further development, but the first results were promising. They showed that with this applied PRP paradigm, multitasking deficits, even in patients at an early stage of their MS disease course, could be detected [136]. Of course, such tests also face challenges, such as time required to perform the test, staff support and the test device. A follow-up study recently investigated the same dual-task test in an outpatient setting using a tablet [147]. Böttrich et al. could show that the accuracy parameter in tablet-based PRP implementations can be used in neuropsychological assessments to examine dual-tasking abilities in pwMS [147]. Such data are promising for the use of such devices for the diagnosis and monitoring of the MS disease course on a regular basis.

## 4. Collection of Digital Biomarkers

The longitudinal and multidimensional acquisition of digital biomarkers is already possible, and includes the use of smartphone-based apps as well as computer- or tablet-based functional tests and questionnaires.

### 4.1. Smartphones and Smartphone Applications

Smartphones are omnipresent everyday objects, and are usually provided with innovative high-quality nine-axis inertial motion sensors that are able to track motion and position in three-dimensional space [148]. These sensors enable basic measurements, such as acceleration or the calculation of data, to conclude how a person walks or to capture their daily step count; the sensing of geographic position, voice analyses and touchscreen pressure can often be measured, detecting falls, monitoring heart rate or daily activity parameters are further examples of what make smartphones today a more and more health-related product [148]. These features can be used when implementing digital assessment into MS patient care. Various applications (apps) use these sensors and extend them by different, other tests that evaluate the functional systems usually affected by MS (e.g., cognition, vision, mobility and fine motor function of the upper limbs). Part of this data collection is done actively, with pwMS performing specific assessments; passive data collection is also possible. Therefore, the pre-installed smartphone developer’s own app (e.g., Health for iOS, Google Fit for Android, etc.) can be used in MS as well for monitoring, e.g., gait. When implementing such apps in research or clinical practice, the precision and accuracy of these sensors need to be considered [96]. To actively collect data from pwMS they need to be prompted either to perform a test or to fill out a questionnaire. Currently, several apps are available that offer a set of various functional tests. Apps such as Floodlight (Roche, Switzerland), Konectom^(TM)^ (Biogen, Cambridge, MA, USA), MSCopilot^®^ (Ad Scientiam, Paris, France) or MS Sherpa (Orikami, Nijmegen, The Netherlands), some of which are still in evaluation and only used in research, collect data regarding mobility (2- or 6-min-walk, U-turns, standing still: distance, speed, balance, etc.), cognition (matching symbols: cognitive processing speed), hand motor function (squeezing objects, drawing lines: coordination, pressure, speed and accuracy of hand and finger movement) and mood (questionnaires), or leaving options for patients to make notes [149,150,151,152]. The benefit of such apps lies in the collection of data from daily life, the possibility to perform functional tests independent of clinical visits, enabling patients to use them for self-evaluation or in cases where they feel as though they are experiencing a worsening of symptoms and, of course, for pwMS and treating neurologists in order to include these data in therapy decisions as well. A regular functional system that monitors and thus detects progression early can lead to early treatment decisions or treatment changes.

The implementation of such apps could overcome the challenge of often infrequent and rare clinic visits and capture all, sometimes daily, even subtle symptom changes. Thus, a more accurate monitoring of the individual disease course and associated optimized therapeutic decisions becomes achievable [66,148,150]. Furthermore, daily patient self-made tasks via a smartphone may contribute to more disease responsibility and informed discussions in clinical visits about subsequent therapeutic steps. PwMS acquire a more active, responsible part of progression monitoring, which might contribute to increased compliance. Adherence to the use of such apps is crucial to allow for longitudinal monitoring, especially in chronic diseases as MS, and will be a challenge.

Other smartphone apps that belong to the group of digital health applications (DIGAs) focus more on special symptoms and can already be prescribed in Germany. These apps aim, e.g., to help and support pwMS regarding fatigue (e.g., elevida by GAIA AG, Germany) and offer talks, exercises and informative material to enable help for self-help, independent of MS management. More DIGAs are already available for patients suffering from anxiety, depression, diabetes, stroke, etc. [153].

### 4.2. Digital Questionnaires in MS

Besides responsible MS management from the side of clinical staff, the patient’s point of view, including quality of life together with subjective treatment and disease effects, is increasingly weighted and raised via PROMs. PROMs combine any information “of a patient’s health condition that comes directly from the patient, without interpretation of the patient’s response by a clinician or anyone else” [154], allowing the specification of whether patients’ feelings/thoughts are congruent with those of clinicians [1,155,156]. With the aim of patient-centered therapeutic management, PROMs are collected directly from patients and contain items that subjectively rate functioning/activity limitations, symptoms, quality of life and health-related quality of life [157,158]. Existing PROMs focus on patients’ subjective evaluation of dealing with fatigue (e.g., Fatigue Assessment Scale), depression (e.g., Hospital Anxiety and Depression Scale), quality of life (e.g., NeuroQoL), mobility (e.g., 12-Item Multiple Sclerosis Walking Scale) and many more. To date, few PROMs of sufficient psychometric quality are available, necessitating the development of standardized, high-quality MS-specific PROMs to collect robust, consistent and reliable real-world data [156,159]. Additionally, electronically answered questionnaires via app-based technologies such as tablets/smartphones, or via the Internet, enable more frequent PROM collection even in-between clinical visits, allowing a closer patient-centered view. Combined with a transmission of patients’ answers into an electronic health record system, it could function as an automated monitoring/notification system in the case of concerning symptoms [1,160]. To avoid long and burdensome questionnaires it would be desirable that PROMs become adaptive to each individual person with MS. The use of computerized adaptive testing is based on an item response theory to decrease administration time, still maintain accuracy, diminish the floor and ceiling effect and also improve the ability to detect the minimal clinically significant difference among patients [161,162,163]. This would lead to a higher patient adherence to perform PROMs on a regular basis, as well as more precise and individualized outcomes.

### 4.3. Digital Data Collection in MS

As there is already the possibility of data collection in many ways and areas, these data are of no use if they cannot be centrally stored, analyzed and made available to healthcare professionals and even patients. Especially when we think of the collection of big data to pave the way to a personalized treatment and consider MS as a lifelong disease that needs thorough and regular monitoring, quality care should enable a digitally supported quick response to any kind of disease worsening. Therefore, the Multiple Sclerosis Documentation System (MSDS) project group started to develop the MSDS software with the support of the Hertie Foundation in 1999, followed by the integrative patient management system MSDS3D, adapting to growing data collection and documentation needs [164]. The integration of a survey system for questionnaires, which not only can be made available on tablets while pwMS come to their visits but can also be sent by email with regular reminders, and thus immediately be documented in patients’ medical records and visible for HCPs, became another feature of MSDS in recent years [165]. Documentation of medication plans, comedication and comorbidities, EDSS and relapses as well as pre-defined procedures for pwMS on a certain DMT, or even without any therapy, also support the monitoring and follow-up of meeting quality standards, and provide hints for improving medical care in the future. As pwMS are not only treated by neurologists alone, but by a variety of other HCPs, such as neuroradiologists, general practitioners, dermatologists, nursing services, psychiatrists, pain management therapists, etc., the integration of interfaces for using telemedicine services, digital communication and sharing medical data with patients, practitioners and caregivers play an increasingly crucial role in MS care [165]. These big data create a holistic picture of an individual patient and lead to specific therapy decisions. Additionally, from an economic point of view to avoid duplicate examinations and to enable high-quality treatment, all these data need to be exchanged between the parties involved. In the future, this need must be further met to provide holistic, high-quality and personalized care to pwMS.

### 4.4. Magnet Resonance Imaging

MRI scans are a standard investigation in MS and are essential both for diagnosis and as a monitoring tool, and are already documented digitally. MRI, in general, cannot only assist in the diagnostic process but is also crucial in regular monitoring to provide information about the treatment response as well as the efficacy and safety of DMTs [166]. Software systems that assist neuroradiologists in evaluating MRIs are already used in clinical trials and investigated regarding their ability to support neuroradiologists and enhance the evaluation of imaging. They can scan defined MRI sequences for the quantification of new or enlarging lesions, lesion volume and brain atrophy. Different companies are working on such software systems, which are partly already used in regular care [165]. Here, an inter-scanner reproducibility is of great importance [167]. Efforts have been made to provide consensus guidelines on the use of MRI in pwMS [168]. This is beyond this review on clinical, digital biomarkers in MS.

### 4.5. The Future of Digital Biomarkers

Much research is in progress regarding digital biomarkers in MS and other diseases, and studies are already evaluating the use of various devices for their collection (Konect-MS and Floodlight), those already available as DIGAs (elevida) or those about to be one (MS Sherpa, Emendia MS). Chronic diseases such as MS or Alzheimer’s disease are complex and can show a diversity of symptoms. These symptoms can also emerge in other diseases, e.g., depression (speech and cognition). Therefore, there will not be “the ideal” digital biomarker with which a disease can be detected and monitored. Rather, it will be the case that different applications will be used, which can easily be installed on smartphones (MS Sherpa, Floodlight, Konectom, elevida, etc.) or integrated into telemedicine (e.g., speech analysis).

## 5. Data Analysis

The use of digital biomarkers creates different demands on data analysis than the traditional processing of data in everyday clinical practice and even than those on a more elaborate level in clinical trials. To fulfill the predictive purpose of a biomarker, real-time data transmission and analysis is the goal. This requires independence of location and data collection situation, i.e., data processing that can take place in clinical practice, but is not limited to the neurologist’s premises, and the visits that take place at longer intervals. To accomplish this, data from a wide variety of sources must be digitally aggregated via standardized secure interfaces (see Section 4.3)—a task far beyond the capabilities of individual apps.

Isolated analyses can also be performed locally, offline, on individual end devices (e.g., the calculation of individual PROM scores) and, assuming timely transmission to the treating neurologist, fulfill targeted warning functions. Here, the general requirement for (automated) information processing systems is that they can reliably distinguish useful information (real medical needs) from noise, such as by applying established cut-off values. These are usually predefined values, which are usually applied population-wide, and the exceeding of which is associated with the presence of an indication. However, the full potential of digital biomarkers as part of a precision medicine approach can only be accessed by integrating a wide variety of data sources into an electronic repository. This is based on the insight that single biomarkers can hardly be used to control a disease as complex as MS, the disease activity of which is, to a large extent, pre-symptomatic, and that rigid, generalized thresholds often do not best reflect the individual situation.

The aim of an integrative evaluation of digital biomarkers in combination with other (clinical) data sources is the creation of a valid statistical model which evaluates prognostic tasks, such as selection and change recommendation regarding a DMT, as well as retrospective processing of information on progression assessment, therapy efficacy and safety aspects, and makes them applicable to individual cases. On the one hand, this results in the necessity of the highest possible data density with regard to the data diversity and the temporal distribution of the surveys. On the other hand, the requirements for the analysis also make it clear that this cannot be achieved with traditional statistical methods/models. The now-established solution for such concerns is found in the field of machine learning. Here, complex data structures are evaluated in a data-driven manner, and information of various types is processed jointly. The desired application situation in real-time and prognostic performance of the model can be extended by self-optimizing methods of deep learning. Schwab et al. chose an application situation for MS for this purpose, in which they aimed to achieve (retrospective) classification between pwMS and healthy controls by evaluating digital biomarkers from smartphone data using deep learning [169]. While this was not yet done as part of an established multiprofessional digital infrastructure for MS, they were able to successfully incorporate multi-layered data on mobility, upper limb functionality, cognition and affect.

However, the further the performance of such an analysis system goes, the more its ability to make recommendations and prognostic deductions comes into focus. This begins with immediate predictions of the general state of impairment from a current cross-sectional measurement of a patient [170], and increases through the consideration of individual longitudinal courses to the prediction of individual symptom areas and the competing effectiveness of therapies. At the same time, this increases the regulatory requirements for digital analysis systems for clinical practice, which in Germany, for example, are regulated by the Medical Devices Act. The end product of integrated digital data analysis is, in the best case, an approved product, which can be used by different HCPs as well as by the individual patient for recording as well as for evaluation, which remains self-updating on the best scientific level and derives understandable as well as useful parameters and overviews for all parties involved.

## 6. Digital Twins

Digital biomarkers are an important component of so-called digital twins. A digital twin in healthcare is a virtual copy of a patient that exactly matches that patient’s characteristics and attributes, thus mirroring that patient. Using machine learning algorithms, the digital twin can be trained to predict disease progression and simulate treatments without risk to the patient. This involves using population data collected from previous patients and study cohorts to build and validate statistical and mechanistic models and to create a population-based digital twin, as well as analyzing data from the individual patient using the existing models and, in turn, integrating them into the patient’s digital twin. The comparison and interaction between the digital twins provide valuable insights (e.g., phenotyping, risk assessment and the prediction of disease evolution) that are clinically interpreted and combined with traditional data to support clinical decision-making. In the process, the digital twin is constantly fed with new data so that it adapts and continuously improves [171]. To create the digital twin of a patient, a large and multidimensional amount of data is needed. A digital twin for MS (DTMS), due to the complexity and long-term nature of the disease, requires a particularly large and multidimensional amount of high-quality, high-frequency and structured data to propose a tailored therapy for the patient. These data are, in detail, physiological condition data of the patient (structured clinical data, paraclinical and multimicrobial data as well as patient-reported data) and procedures applied to the patient (diagnostic workup, treatment and monitoring, integrated in personalized clinical pathways). Many clinical and paraclinical data, including lab and imaging data, can be captured with digital biomarkers that can be transformed into interpretable outcome measures using algorithms. Digital twins also offer the possibility of visualizing a wide variety of parameters using a dashboard and mapping personalized clinical pathways. With the development of a DTMS, clinical treatment decisions, physician–patient communication and thus the quality of treatment can be improved. Even though there are still many challenges to be overcome on the way to the DTMS (effectiveness and safety, data protection, data security, data quality data management, creation of meaningful algorithms and ethical as well as individual concerns) and a DTMS need to be validated and tested before being used in practice, it is a valuable tool with which to make precision medicine and patient-centered care in MS part of everyday clinical practice [172].

## 7. Conclusions/Summary

The heterogeneous, multisymptomatic MS disease offers numerous possibilities for the acquisition of digital biomarkers. As the possibilities to collect digital data are continuously growing, such data can also be used for prognostic and diagnostic aspects as well as for the evaluation of disease activity and response to therapy. These digital biomarkers can be collected by devices available to everyone (e.g., wearables such as fitness trackers) or special devices created for specific examinations (e.g., vision, upper and lower limb function, MRI, cognition, PROMs, etc.). Therefore, they need to be validated, standardized, analyzed and made available to HCP to be used in pwMS care.

To our knowledge, older MS patients are becoming more and more familiar with using new technologies such as apps on smartphones or tablets [164]. Additionally, the in-clinic collection of digital biomarkers by a physician or escorting staff benefits all patients, regardless of their age.

As MS is a lifelong disease, pwMS should be integrated into their treatment. Here, smartphone applications can be used to document mood or specific problems (e.g., headache, fatigue, depression, etc.) or to check functional systems on a regular basis (such as vision, cognition, motor function of the extremities, etc.). The use of digital biomarkers may also be of interest to developing countries, where medical/neurological care is not widely available. For example, data could be collected from patients and transmitted to physicians as soon as Internet access is available, or a voice analysis could be performed via a telephone call. However, the establishment and validation procedures of digital biomarkers do not yet follow generally accepted standards. Developments according to the requirements of the Medical Devices Act are necessary, but are as complex as the development of classical biomarkers. Once the collection of standardized, validated digital biomarkers in all aspects of life (in-clinic and in daily life) is possible, the way is clear to develop digital twins and personalized treatment.

## Figures and Tables

**Table 1:** Challenges in implementing digital biomarkers in the clinic.

Benefits	Challenges
Continuous real-time data	Privacy
Better real-world evidence	Adherence/retention
Greater power	High variability
Novel, sensitive endpoints	Validation required
Faster decisions	Complex analysis
Big data	Data storage

**Table 2:** Advantages and disadvantages of various gait assessment methods (AmI = ambulation index; EDSS = Expanded Disability Status Scale; T25FW = Timed 25-Foot Walk test; 6MWT = 6-Minute Walk Test; MSWS-12 = 12-item Multiple Sclerosis Walking Scale; and EMIQ = Early Mobility Impairment Questionnaire) [73,74,75].

	Outcome Measures	Advantages	Disadvantages
Standardized clinical measures.	- Disability score (EDSS). - Time and degree of assistance required to walk 25 feet.	- Take into account the use of assistive devices. - EDSS: directly related to neurologic examination; used in clinical trials. - AmI: simple and quick.	- Require a skilled examiner. - Do not identify mechanisms underlying gait dysfunction. - EDSS and AmI have limited precision and responsiveness. - No normative data.
Timed measures (e.g., T25FW, 6MWT).	Quantified aspect of gait, such as speed and endurance.	- Simple. - Readily quantified. - Require limited training. - Published norms available.	Do not identify mechanisms underlying gait dysfunction.
Patient-based measures (e.g., MSWS-12; EMIQ).	Patient’s perspective of their walking disability.	- Document the patient’s perspective. - Require little time to complete.	Do not identify mechanisms underlying gait dysfunction.
Observational gait analysis (e.g., during T25FT or other walking conditions).	Gait pattern in terms of kinematic and spatiotemporal parameters.	- Identify mechanisms underlying gait dysfunction. - Requires limited time and equipment.	- Limited validity, reliability and precision. - Requires skilled examiner.
Sensor floor plates: (a) Instrumented walkways; (b) Force platform; (c) Balance boards.	(a) Spatial and temporal variables. (b) Ground reaction force pattern. (c) Kinematics. (d) Ground reaction force pattern.	(a) Simple. - Clinical feasibility. - Objectivity. - Quantification. - Good sensitivity. (b) Objectivity. - Quantification. - Good sensitivity. (c) Objectivity. - Quantification. - Portability.	(a) Require equipment. - Do not identify mechanisms underlying gait dysfunction. - Restricted to clinic or laboratory environments. - Restricted to few steps at a time. (b) Restricted to laboratory environments. (c) Clinical, research and home.
Three-dimensional gait analysis (reflecting markers places on a person and recording movement with infrared cameras).	Detailed quantitative measures of kinematic, kinetic and spatiotemporal parameters.	- Identify mechanisms underlying gait dysfunction. - Provide precise kinematic, kinetic, and spatiotemporal data.	Require expensive equipment and skilled examiner.
Video-based: (a) Marker-based motion capture; (b) Marker-free motion capture.	(a) and (b): - Spatial and temporal variables. - Kinematics. - Joint range of motion.	(a) Comprehensive analysis of the widest range of gait variables. - Power consumption is not an issue. - Little interference from external environmental factors. (b) Objectivity. - Quantification. - High sensitivity. - Comprehensiveness. - Better suited to clinical environments than marker-based systems.	(a) Expensive. - Must be used in a laboratory environment. - Markers and restricted space can hinder movement. (b) Can be expensive. - Generally, cannot be used outside the clinic or laboratory environment. - Measures a restricted number of steps.
Wearable sensors: (a) Inertial sensors (research-oriented/consumer-driven); (b) Pressure sensors.	(a) Spatiotemporal measures: - Joint range of motion. - Kinematics. - Balance. (b) Spatial and temporal variables.	(a) Clinical feasibility. - Objectivity. - Quantification. - Good sensitivity. - Face validity. (b) Clinical feasibility. - Objectivity. - Quantification. - Good sensitivity. - Can be used outside the clinic and laboratory.	(a) Sensors can impede movement. - Battery power. - Susceptible to environmental interference. - May need technical operators. (b) Sensors can impede movement. - Battery power.

**Table 3:** Potential gait assessment technologies in MS.

Assessment Technology	Method	Outcomes *	Device ^ǂ^ (Manufacturer)
Video-based	(a) Marker-based (b) Marker-free	(a) Joint range of motion	(a) Vicon (Civon Motion Systems Ltd.); Miqus Hybrid (Qualisys AB) (b) Miqus Hybrid (Qaulisys AB)
Sensor floor plates	(a) Instrumented walkway (b) Force platform (c) Balance boards	(a) Spatiotemporal measures (b) Ground reaction force pattern (c) Ground reaction force pattern	(a) GAITRite (CIR Systems) (b) ProKin (Tecnobody); 3D Force Plate (Kistler Instruments AG (c) Wii Balance Board (Nintendo)
Wearable sensors	(a) Research-oriented ^±^ (b) Consumer-driven ^ƍ^	(a) Spatiotemporal measures, joint range of motion	(a) Mobility lab (APDM), XActiGraph GT9X Link (ActiGraph); GENEActiv Original (Activinsights) (b) Fitbit Charge 5 (fitbit), vívosport^®^ (Garmin), Xiaomi Mi Band 6 (Xiaomi)

* Selection of key outcomes; ^ǂ^ examples; ^±^ devices developed primarily for research purposes: no direct patient feedback, no modifying of movement behavior through, e.g., motivation, raw data output; ^ƍ^ devices developed primarily for consumer requirements: direct feedback of movement behavior on device display, no direct access to raw data (adapted from Trentzsch et al., 2020 [74]).
